# The quality of life of comorbid patients with pathology of thyroid gland and gastroesophageal reflux disease

**DOI:** 10.1192/j.eurpsy.2021.651

**Published:** 2021-08-13

**Authors:** M. Artemieva, I. Manyakin, E. Basova, V. Fon Ratenau

**Affiliations:** Psychiatry And Medical Psychology, RUDN University, Moscow, Russian Federation

**Keywords:** quality of life, pathology of thyroid gland, comorbidity, gastroesophageal reflux disease

## Abstract

**Introduction:**

In Russia, the prevalence of GERD is 18-46% [Ivashkin V. T., Maev I. V., Trukhmanov A. S., 2011]. GERD leads to a significant decrease in the quality of life of patients, especially with nocturnal symptoms, extraesophageal symptoms (chest pain, persistent cough), and increases the risk of complications such as bleeding from ulcers and erosions, peptic strictures and, which causes the greatest caution, Barrett’s esophagus and esophageal adenocarcinomas

**Objectives:**

The medical and social significance of the pathology of the thyroid gland and gastrointestinal tract problem is determined by their high prevalence regardless of age, the annual increase in morbidity and the decrease in the quality of life (QOL).

**Methods:**

Patients were divided in two groups: patients with GERD; patients with GERD and hypothyroidism. Quality of life was studied using the non-specific (general) SF 36 questionnaire.

**Results:**

The lowest indicators were shown by patients with GERD and hypothyroidism (PF scale - Me 75.00 and 45.00 (p=0.005470), RF scale - 75,00 and 25.00 (p=0.043046), BP scale - 74.00 and 52.00 (p=0.036493), GH scale - 58.00 and 15.00 (p=0.009959)). The second group was more disadvantaged (VT scale - IU 52.50 and 32.50 (p=0.098125), SF scale - 75 00 and 50.00 (p=0.019016), RE scale - 33.30 and 0.00 (p=0.028841), GH scale - 48.00 and 36.00 (p=0.025919).

**Conclusions:**

According to the results, the presence of combined pathology of the thyroid gland and gastrointestinal tract significantly affected the physical and psychological component of health in the studied patients.

